# Bone geometry of the hip is associated with obesity and early structural damage – a 3.0 T magnetic resonance imaging study of community-based adults

**DOI:** 10.1186/s13075-015-0631-4

**Published:** 2015-04-30

**Authors:** Andrew J Teichtahl, Yuanyuan Wang, Sam Smith, Anita E Wluka, Michael Zhu, Donna Urquhart, Graham G Giles, Richard O’Sullivan, Flavia M Cicuttini

**Affiliations:** Department of Epidemiology and Preventive Medicine, School of Public Health and Preventive Medicine, Monash University, Alfred Hospital, Melbourne, VIC 3004 Australia; Baker IDI Heart and Diabetes Institute, Commercial Road, Melbourne, VIC 3004 Australia; Centre for Epidemiology and Biostatistics, Melbourne School of Population and Global Health, The University of Melbourne, Carlton, VIC 3053 Australia; Cancer Epidemiology Centre, Cancer Council Victoria, Melbourne, VIC 3004 Australia; MRI Department, Healthcare Imaging Services, Epworth Hospital, Richmond, VIC 3121 Australia; Department of Medicine, Central Clinical School, Monash University, Melbourne, VIC 3004 Australia

## Abstract

**Introduction:**

The mechanism by which obesity increases the risk of hip osteoarthritis is unclear. One possibility may be by mediating abnormalities in bony geometry, which may in turn be associated with early structural abnormalities, such as cartilage defects and bone marrow lesions.

**Methods:**

One hundred and forty one older adults with no diagnosed hip osteoarthritis had weight and body mass index measured between 1990 and 1994 and again in 2009 to 2010. Acetabular depth and lateral centre edge angle, both measures of acetabular over-coverage, as well as femoral head cartilage volume, cartilage defects and bone marrow lesions were assessed with 3.0 T magnetic resonance imaging performed in 2009 to 2010.

**Results:**

Current body mass index, weight and weight gain were associated with increased acetabular depth and lateral centre edge angle (all *P* ≤ 0.01). For every 1 mm increase in acetabular depth, femoral head cartilage volume reduced by 59 mm^3^ (95% confidence interval (CI) 20 mm^3^ to 98 mm^3^, *P* < 0.01). Greater acetabular depth was associated with an increased risk of cartilage defects (odds ratio (OR) 1.22, 95% CI 1.03 to 1.44, *P* = 0.02) and bone marrow lesions (OR 1.29, 95% CI 1.01 to 1.64, *P* = 0.04) in the central region of the femoral head. Lateral centre edge angle was not associated with hip structure.

**Conclusions:**

Obesity is associated with acetabular over-coverage. Increased acetabular depth, but not the lateral centre edge angle, is associated with reduced femoral head cartilage volume and an increased risk of cartilage defects and bone marrow lesions. Minimising any deepening of the acetabulum (for example, through weight management) might help to reduce the incidence of hip osteoarthritis.

## Introduction

Although not as strongly or consistently reported at the knee, obesity is a recognised modifiable risk factor for hip osteoarthritis (OA) [1-7]. The exact mechanism by which obesity mediates OA at weight-bearing joints such as the hip is unclear. Variations in the shape of bone at the hip are thought to be important in the pathogenesis of hip OA [8-11]. Added load imparted by obesity may be important for bony remodelling, as has been exemplified by the association between increased body mass index (BMI) and metaphyseal bone expansion at the proximal tibial plateau [12]. It is well established that remodelling of bone in response to a load occurs via complex mechanotransduction mechanisms. These are processes whereby mechanical signals are converted via cellular signalling to biochemical responses [13].

An example of abnormal hip bone geometry contributing to joint pathology is femoroacetabular impingement (FAI), a cause of premature hip OA [14]. FAI occurs when either a non-spherical femoral head compacts the acetabular rim (cam deformity) [15], or acetabular over-coverage limits the range of movement of the hip joint (pincer deformity) [9] or when cam and pincer deformities co-exist. In these instances, abnormal bone geometry between the femoral head and acetabulum is thought to mechanistically account for cartilage damage [16,17]. Whether the association between obesity and risk of hip OA might be partly explained by an influence on bone geometry has not previously been examined.

It is also unclear whether variations in bone geometry are associated with deleterious changes in hip joint structures in older adults. Acetabular over-coverage has commonly been assessed by two measures: acetabular depth and the lateral centre edge angle (LCEA) [16,18]. These measures are thought to capture hip joint incongruency. While a magnetic resonance imaging (MRI) study reported greater acetabular depth to be associated with cartilage lesions [16], a radiographic study detected no association between the LCEA and the risk of incident radiographic hip OA over 5 years [18]. Notably, a 13% mean reduction in femoral head cartilage volume is demonstrable before any evidence of radiographic joint space narrowing [19]. Thus, radiographs may not be sufficiently sensitive to assess the association between hip bone geometry and early structural abnormalities at the hip, such as the presence of cartilage defects and bone marrow lesions (BMLs), which have been associated with clinical outcomes including radiographic hip OA [20-22].

The aim of this MRI study was to examine whether measures of acetabular over-coverage were associated with obesity and structural abnormalities in older adults with no diagnosed hip OA.

## Methods

### Participants

One hundred and forty one participants with no diagnosed hip OA were recruited as previously described [23] from the Melbourne Collaborative Cohort Study (MCCS), a prospective cohort study of residents of Melbourne, Australia, aged 40 to 69 years at MCCS inception (1990 to 1994) examining healthy aging [24]. There were no significant differences between this population and the original population, which had the following profile: 61% women, mean (standard deviation) age 57.8 (3.0) years and BMI 25.7 (3.8) kg/m^2^ at baseline (1990 to 1994). Participants were eligible for the current study if they were aged 50 to 85 years without any of the following exclusion criteria: a diagnosis of hip OA from a medical or allied health professional; significant hip pain lasting for >24 hours in the last 5 years; a previous hip injury requiring non-weight bearing treatment for >24 hours or surgery (including arthroscopy); or a history of any form of arthritis diagnosed by a medical practitioner. Any person was further excluded if they had any malignancy or any contraindication to MRI, including a pacemaker, metal sutures, presence of shrapnel or iron filings in the eye, or claustrophobia. The study was approved by the Human Research Ethics Committees of Cancer Council Victoria and Monash University. All participants gave written informed consent.

### Anthropometric data

Height and weight were measured at MCCS inception (1990 to 1994) and approximately 16.9 (±0.61) years later at the time of MRI (2009 to 2010). Weight was measured to the nearest 100 g using digital electronic scales. Height was measured to the nearest 1 mm using a stadiometer and a metal anthropometric tape. BMI was calculated as the weight in kilograms divided by the square of height in meters. Percentage weight change was calculated by (follow-up weight (kg) – baseline weight (kg))/baseline weight (kg) and multiplied by 100.

### Magnetic resonance imaging measurements

Each participant had an MRI scan performed on the dominant hip (defined by the leg used to kick a ball) in 2009 to 2010. The hip joint was imaged on a 3.0-T whole body magnetic resonance unit (Siemens, Verio, Siemens Medical, Germany) using a phased array flex coil. Sagittal images were obtained using a T_2_-weighted fat-suppressed three-dimensional gradient-recalled acquisition sequence in the steady state (repetition time 14.45 milliseconds, echo time 5.17 milliseconds; flip angle 25°, slice thickness 1.5 mm, field of view 16 cm, pixel matrix 320 × 320, acquisition time 7 minutes 47 seconds, and 1 acquisition). Coronal images were obtained using a fat saturation, proton density, spin echo acquisition sequence (repetition time 3,400 milliseconds, echo time 64 milliseconds, flip angle 90°, slice thickness 3 mm, field of view 16 cm, pixel matrix 256 × 256, acquisition time 5 minutes 26 seconds, and 1 acquisition). A musculoskeletal radiologist (YW) with over 15 years experience using structural outcomes determined by MRI in epidemiological studies supervised and independently monitored measurements. One observer, trained by the radiologist, was responsible for measuring one structural outcome (for example, cartilage volume, cartilage defects or BMLs). Each observer was also required to assess their designated structural measure in duplicate, at least 1 week apart and blinded to their previous assessment and characteristics of the participants.

**Figure 1:**
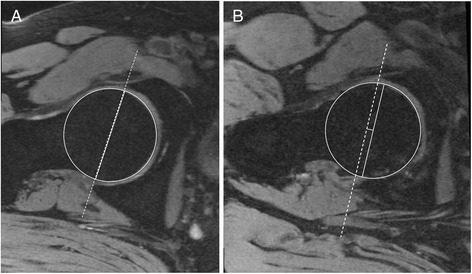
Acetabular depth assessed in the axial plane. **(A)** Neutral - acetabular rim in line with centre of femoral head. **(B)** Acetabular over-coverage - centre of femoral head translated medially relative to acetabular rim.

Acetabular depth was assessed from MRI using a previously described method [16]. The transverse oblique image was obtained through the centre of the femoral neck. The acetabular depth was measured as the distance between the centre of the femoral head and the line connecting the anterior acetabular rim to the posterior acetabular rim. The value was positive if the centre of the femoral head was medial to the line connecting the acetabular rims (that is, acetabular over-coverage) (see Figure 1). The intra-observer reproducibility (assessed by intra-class correlation coefficient; ICC) was 0.87.

**Figure 2:**
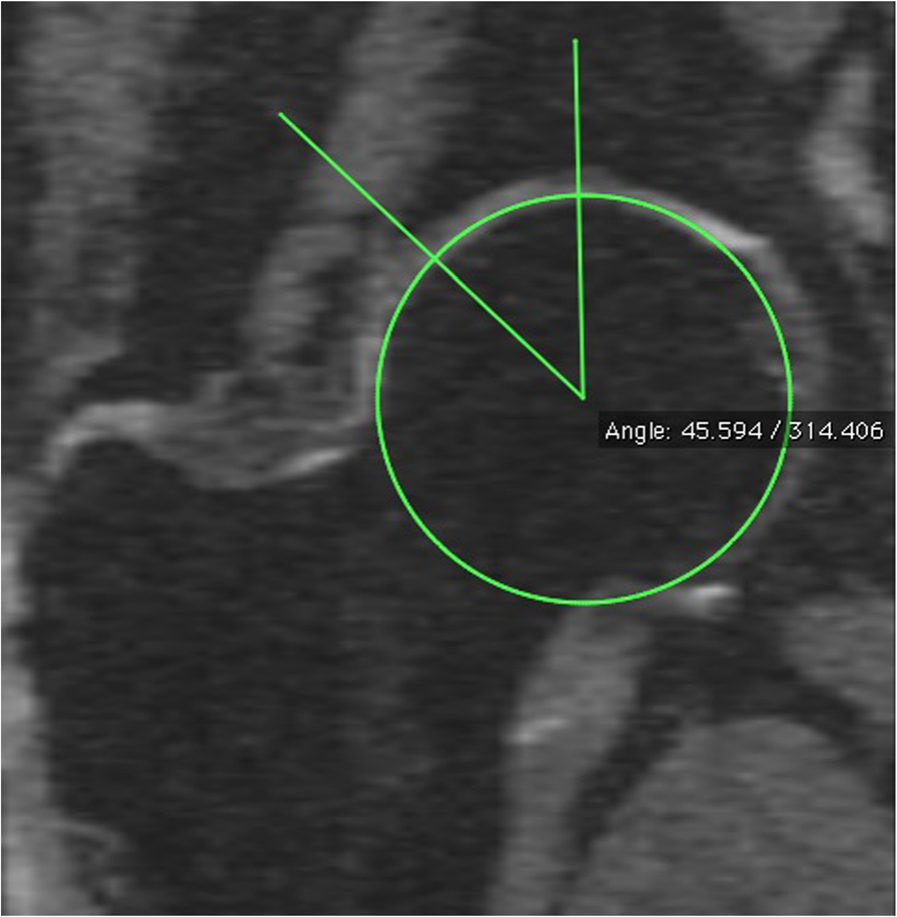
Lateral centre edge angle.

LCEA was measured from a re-formatted coronal image using the same methods as for radiographs [25]. LCEA was the angle formed by the line from the lateral acetabular rim to the central point of the femoral head, and the vertical line through the central point of the femoral head (see Figure 2). The intra-observer reproducibility (assessed by ICC) was 0.87.

Femoral head cartilage volume was measured on T_2_-weighted sagittal images using the software Osiris (version 4.19; Geneva University Hospital, Geneva, Switzerland) as previously described [19]. The image data were transferred to the workstation, and an isotropic voxel size was then obtained by a trilinear interpolation routine. The volume of the femoral head cartilage was isolated from the total volume by manually drawing disarticulation contours around the cartilage boundaries on each image section. These data were then re-sampled by bilinear and cubic interpolation for the final three-dimensional rendering. The volume of the femoral head cartilage was determined by summing all the pertinent voxels within the resultant binary volume. Femoral head cartilage volume was measured in duplicate with at least a 1-week interval by one trained observer. The coefficient of variation was 2.5% [19]. The intra-observer reproducibility (assessed by ICC) was 0.99.

**Figure 3:**
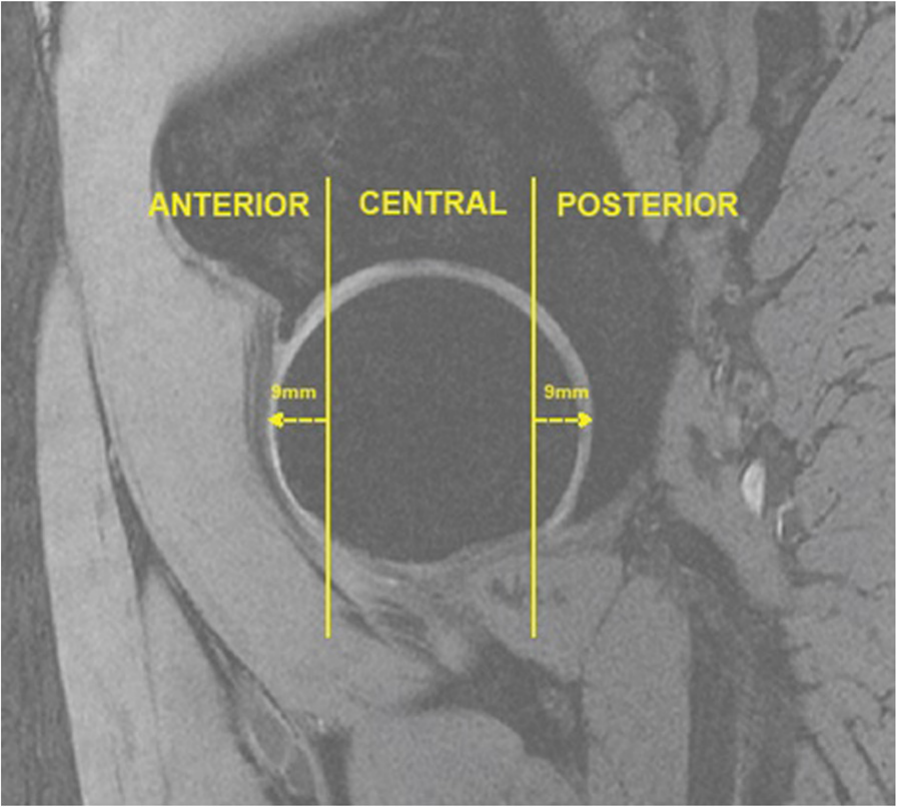
Regional zones of the hip joint.

The femoral head was divided into three regions (central, anterior and posterior) to assess cartilage defects and BMLs. The anterior and posterior regions were assessed in the sagittal plane and corresponded to the first and last three coronal slices (9 mm) (Figure 3). The area in between the anterior and posterior region was termed the central region. The division of anterior, central and posterior regions was adapted from methods used in previously published works [21,22].

Femoral head cartilage defects were assessed from proton density coronal images and confirmed on sagittal imaging for the central region, and from the sagittal imaging for the anterior and posterior regions. The presence of cartilage defects was defined as a grade 3 (that is, deep ulceration with 50% loss of thickness or greater) or 4 defect (full-thickness chondral wear with exposure of subchondral bone) on at least two consecutive slices as previously described [26]. The intra-observer reproducibility (assessed by ICC) was 0.72 for cartilage defects.

**Figure 4:**
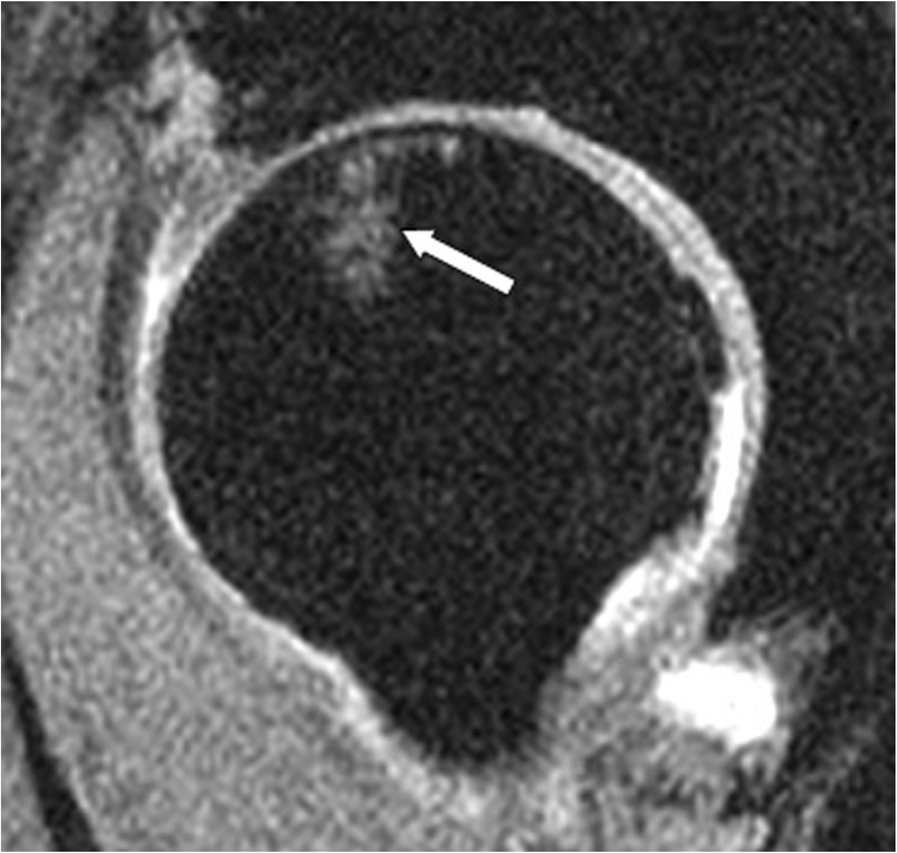
An example of a central bone marrow lesion visualised from the sagittal image.

BMLs were said to be present if high-intensity signal was apparent in the subchondral region (Figure 4) on two or more consecutive slices. One trained observer, who was blinded to participant’s characteristics, assessed the presence of BMLs for each participant in duplicate, at least 1 week apart. The intra-observer reproducibility (as assessed by ICC) was 0.94.

### Statistical analyses

Multiple linear regression analyses were used to determine the relationship between acetabular depth/and LCEA and obesity measures, as well as femoral head cartilage volume. Binary logistic regression was used to determine the relationships between acetabular depth and LCEA and the risk of both femoral head cartilage defects and BMLs. All analyses were adjusted for age, gender and BMI. A *P*-value of less than 0.05 (two-tailed) was regarded as statistically significant. All analyses were performed using SPSS statistical package (standard version 20.0; SPSS, Chicago, IL, USA).

## Results

Subject characteristics are shown in Table 1. The cohort was predominantly female (56%) and aged on average 66.7 (±7.4) years.

**Table 1:** **Subject characteristics (n = 141)**

**At magnetic resonance imaging (2009 to 2010)**	
Age (years)	66.7 (7.4)
Gender (female) (n (%))	79 (56)
Weight (kg)	76.1 (15.1)
Body mass index (kg/m^2^)	27.6 (4.8)
Acetabular depth (mm)	-3.69 (2.37)*
Lateral centre edge angle (degrees)	36.8 (6.7)**
Femoral head measures	
Cartilage volume (mm^3^)	3317 (749)
Cartilage defects (n (%))	
Central	88 (62.4)
Anterior	5 (3.5)
Posterior	25 (17.6)
Bone marrow lesions (n (%))	
Central	13 (9.2)
Anterior	3 (2.1)
Posterior	4 (2.8)
**Obesity measures (1990 to 1994)**	
Weight (kg)	72.9 (13.7)
Body mass index (kg/m^2^)	26.0 (4.0)
**Change (1990-1994 to 2009-2010)**	
Percentage weight gain	4.7 (9.6)

Results displayed as mean (standard deviation) unless otherwise stated. *Range -9.9 mm to 5.9 mm. **Range 15.2 to 59.4 degrees.

The associations between measures of obesity and acetabular over-coverage measures are shown in Table 2. Increasing acetabular depth was associated with increased BMI (β = 0.22 mm, 95% confidence interval (CI) 0.05 to 0.38 mm, *P* < 0.01) and body weight (β = 0.08 mm, 95% CI 0.02 to 0.14 mm, *P* = 0.01) after adjusting for potential confounders. Similarly, increased BMI (β = 0.69°, 95% CI 0.22 to 1.16°, *P* < 0.01) and body weight (β = 0.23°, 95% CI 0.06 to 0.40°, *P* < 0.01) were associated with an increased LCEA after adjusting for potential confounders. For each 1% gain in weight between 1990 to 1994 and 2009 to 2010, there was a 0.06-mm increase (95% CI 0.01 to 0.10 mm) in acetabular depth and a 0.16° increase (95% CI 0.04 to 0.28°) in the LCEA. We also performed these analyses based on gender subgroups. Most of these associations remained statistically significant for females only. Although not statistically significant, the magnitude and direction of results was, however, similar for males.

**Table 2:** **The associations between acetabular over-coverage measures and obesity measures**

	**Total population (n = 141)**				**Males (n = 62)**		**Females (n = 79)**	
	**Univariate analyses**	***P***	**Multivariate analyses**	***P***	**Multivariate analyses**	***P***	**Multivariate analyses**	***P***
**Acetabular depth**								
**2009-2010**								
BMI (kg/m^2^)^a^	0.05 (-0.03, -0.14)	0.20	0.22 (0.05, 0.38)	<0.01	0.17 (-0.18, 0.52)	0.33	0.27 (0.09, 0.46)	<0.01
Weight (kg)^b^	-0.01 (-0.04, 0.02)	0.47	0.08 (0.02, 0.14)	0.01	0.05 (-0.06, 0.16)	0.36	0.10 (-0.03, -0.18)	<0.01
*Change (1990-1994 to 2009-2010)*								
Percentage weight gain^c^	0.06 (0.01, 0.10)	<0.01	0.06 (0.01, 0.10)	<0.01	0.05 (-0.05, 0.14)	0.32	0.07 (0.02, 0.11)	<0.01
**Lateral centre edge angle**								
**2009-2010**								
BMI (kg/m^2^)^a^	0.18 (-0.06, 0.41)	0.14	0.69 (0.22, 1.16)	<0.01	0.39 (00.41, 1.19)	0.33	0.59 (0.02, 1.17)	0.04
Weight (kg)^b^	0.01 (-0.07, 0.08)	0.81	0.23 (0.06, 0.40)	<0.01	0.11 (-0.15, 0.37)	0.40	0.22 (-0.01, 0.44)	0.06
*Change (1990-1994 to 2009-2010)*								
Percentage weight gain^c^	0.16 (0.05, 0.28)	<0.01	0.16 (0.04, 0.28)	0.01	0.09 (-0.13, 0.31)	0.40	0.15 (0.00, 0.29)	0.04

Results are expressed as β (95% confidence interval). ^a^Multivariate analysis adjusted for gender (for total population analyses), age at magnetic resonance imaging (MRI) and body mass index (BMI) at 1990-1994. ^b^Multivariate analysis adjusted for gender (for total population analyses), age at MRI and weight at 1990-1994. ^c^Multivariate analysis adjusted for gender (for total population analyses), and age at MRI.

The relationships between measures of acetabular over-coverage and femoral head structures are shown in Table 3. In univariate analyses, for every 1-mm increase in acetabular depth, there was an associated 99-mm^3^ reduction in femoral head cartilage volume (95% CI -149 to -48 mm^3^). This relationship remained significant after adjustment for age, gender and BMI (β = -59 mm^3^, 95% CI -98 to -20 mm^3^, *P* < 0.01). Increased acetabular depth was associated with an increased risk of both cartilage defects (odds ratio = 1.22, 95% CI 1.03 to 1.44, *P* = 0.02) and BMLs (odds ratio = 1.29, 95% CI 1.01 to 1.64, *P* = 0.04) in the central region of the femoral head. There were no significant associations between acetabular depth and cartilage defects or BMLs in the anterior or posterior regions of the femoral head (data not shown) and there were no significant associations found between femoral head structures and the LCEA.

**Table 3:** **Association between acetabular over-coverage measures and femoral head structures**

	**Univariate analyses**	***P***	**Multivariate analyses**	***P***
**Acetabular depth**				
Cartilage volume (mm^3^)^a^	-99 (-149, -49)	<0.001	-59 (-98, -20)	0.01
Central cartilage defects^b^	1.20 (1.02, 1.40)	0.03	1.22 (1.03, 1.44)	0.02
Central bone marrow lesions^b^	1.30 (1.03, 1.64)	0.03	1.29 (1.01, 1.64)	0.04
**Lateral centre edge angle**				
Cartilage volume (mm^3^)^a^	-19 (-39, 1)	0.06	-10 (-25, 5)	0.21
Central cartilage defects^b^	1.02 (0.97, 1.07)	0.44	1.02 (0.97, 1.08)	0.41
Central bone marrow lesions^b^	0.99 (0.91, 1.08)	0.89	0.99 (0.90, 1.08)	0.76

^a^Results expressed as β (95% confidence interval). ^b^Results expressed as odds ratio (95% confidence interval). Multivariate analyses adjusted for age (2009-2010), gender and body mass index (2009-2010).

## Discussion

We have demonstrated that increased weight and BMI, as well as weight gain, are associated with acetabular over-coverage, as measured by both acetabular depth and the LCEA. Increased acetabular depth was also associated with reduced hip cartilage volume and an increased risk of cartilage defects and BMLs located in the central region of the femoral head. LCEA was not associated with femoral head cartilage volume, cartilage defects or BMLs.

A previous systematic review concluded that obesity, measured predominantly by an increased BMI, had a moderately positive influence on the development of hip OA, with an odds ratio of approximately 2 [6]. However, it is unclear how obesity might increase the risk of hip OA. Axial load and contact stress through the hip joint may be amplified by obesity, potentially resulting in bony remodelling. To our knowledge, no previous study has examined how obesity might influence bone geometry at the hip. At the knee, an increased BMI was associated with pathological increases in metaphyseal bone expansion at the proximal tibia [12]. In this cross-sectional study, increased acetabular depth and the LCEA, both markers of acetabular over-coverage, were associated with increased obesity measures, and weight gain over approximately 17 years preceding MRI was associated with greater acetabualar over-coverage. In gender subgroup analyses, these associations were statistically significant for females, but not males. Given that the average age of the cohort was 66.7 years, it may be that post-menopausal bones and the concurrent reduction in bone mineral density may make the female hip particularly susceptible to bony remodelling, such as the changes that result in acetabular over-coverage. Bone mineral density studies will help to substantiate this hypothesis.

An important corollary from these data might be the potential for hip OA to be delayed or averted by modifying acetabular over-coverage. We found that an increased acetabular depth was associated with reduced femoral head cartilage volume and an increased risk of cartilage defects and BMLs in the central region of the femoral head. Previously, a smaller study of 50 younger people with confirmed FAI demonstrated that acetabular depth was associated with increased labral cartilage defects [16]. A reduction in femoral head cartilage volume and the presence of cartilage defects have both been associated with radiographic hip OA and outcomes such as self-reported pain and disability [19,21,22]. Hip BMLs have been associated with hip pain and high cartilage signal on MRI [20]. Taken together, these data demonstrate that acetabular depth is associated with structural hip damage and, although requiring confirmation by longitudinal investigation, might be a modifiable predictor of joint damage signifying early hip OA.

Previous studies have failed to demonstrate any associations between joint structure and the LCEA when assessed either intra-operatively [17] or for the development of radiographic hip OA over 5-year follow-up [18]. Nevertheless, radiographic assessment might not be sufficiently sensitive to detect early structural abnormalities at the hip, such as the presence of cartilage defects and BMLs. Consistent with the lack of evidence for the LCEA to be associated with hip joint abnormalities, we found no associations between the LCEA and structural abnormalities within the hip joint using MRI. These results suggest that an increased LCEA does not adversely impact on hip structure, despite its notoriety as a measure of acetabular over-coverage, or pincer deformity. While both acetabular depth and the LCEA may be measuring the same construct (that is, acetabular over-coverage), it appears that acetabular depth rather than the LCEA is related to clinically significant outcomes (that is, structural damage).

This study has several limitations. The relationships between acetabular over-coverage measures and hip structures were examined cross-sectionally and we cannot determine whether an increased acetabular depth precedes hip structural changes, or *vice versa*. For instance, it is unclear whether an increased acetabular depth resulted in reduced cartilage volume, or whether reduced cartilage volume increased the acetabular depth. Longitudinal studies are needed to address this issue. This is also partly the case for obesity measures, although change in obesity was measured over an average of 16.9 years preceding MRI assessment, providing evidence that weight gain might increase acetabular over-coverage. Reverse causality is less likely since participants had not sought medical attention for hip pain, and therefore acetabular over-coverage predating weight gain via activity limitation is unlikely. Radiographs were not performed in this study. Some participants may have had early radiographic OA but MRI measurement of hip cartilage volume, a continuous measure, correlates with radiographic hip OA [19]. Although acetabular depth has not previously been a focus of many studies, it has been validated as a measure of pincer deformity using MRI [16]. Assessment of acetabular depth at two time points would enable associations between changes in obesity and changes in bony geometry to be assessed. In this study, we have examined hip morphometry and have not examined other bone properties such as periarticular bone mineral density, trabeculae structure and bone attrition. It is unlikely that one bone property (for example, shape) changes in isolation without simultaneous changes in other bone properties (for example, attrition), signifying that bone may remodel as a continuum, with changes in several properties. Further studies examining other bone properties are, however, needed to substantiate such claims. Moreover, in this cohort, the prevalence of anterior and posterior femoral head cartilage defects or BMLs was low. Larger studies will help to address such issues and determine whether MRI structural abnormalities outside of the central region are also associated with acetabular over-coverage. The low prevalence of cartilage defects may also be attributable to our conservative method for assessing defects (that is, deep ulceration with 50% loss of thickness or greater) [26], which may have underestimated cartilage defects.

## Conclusions

Increased weight and BMI, as well as weight gain, were all associated with acetabular over-coverage, as measured by both acetabular depth and the LCEA. Greater acetabular depth was also associated with reduced femoral head cartilage volume and an increased risk of cartilage defects and BMLs in the central region of the femoral head. The LCEA was not associated with structural abnormalities at the hip, further questioning the clinical significance of the LCEA as a predictor of hip OA. Although requiring further investigation, reducing acetabular deepening through weight management might help to reduce the incidence of hip OA.

## Abbreviations

BMI: body mass index
BML: bone marrow lesion
CI: confidence interval
FAI: femoroacetabular impingement
ICC: intra-class correlation coefficient
LCEA: lateral centre edge angle
MCCS: Melbourne Collaborative Cohort Study
MRI: magnetic resonance imaging
OA: osteoarthritis
